# Characterization of species-specific genes regulated by E2-2 in human plasmacytoid dendritic cells

**DOI:** 10.1038/srep10752

**Published:** 2015-07-17

**Authors:** Menglan Cheng, Xuyuan Zhang, Haisheng Yu, Peishuang Du, Joël Plumas, Laurance Chaperot, Lishan Su, Liguo Zhang

**Affiliations:** 1 Key Laboratory of Immunity and Infection, Institute of Biophysics, University of Chinese Academy of Sciences, Beijing, China; 2 Department of Research and Development, EFS Rh ône-Alpes Grenoble, La Tronche, France; 3 Lineberger Comprehensive Cancer Center, Department of Microbiology and Immunology, School of Medicine, University of North Carolina at Chapel Hill, Chapel Hill, NC, USA

## Abstract

Dendritic cells (DCs) are sentinels of the immune system and comprise two distinct subsets: conventional DCs (cDCs) and plasmacytoid DCs (pDCs). Human pDCs are distinguished from mouse pDCs phenotypically and functionally. Basic helix-loop-helix protein E2-2 is defined as an essential transcription factor for mouse pDC development, cell fate maintenance and gene programe. It is unknown whether E2-2 regulation contributes to this species-specific difference. Here we investigated the function of E2-2 in human pDCs and screened human-specific genes regulated by E2-2. Reduced E2-2 expression in human pDC cell line GEN2.2 resulted in diminished IFN-α production in response to CpG but elevated antigen presentation capacity. Gene expression profiling showed that E2-2 silence down-regulated pDC signature genes but up-regulated cDC signature genes. Thirty human-specific genes regulated by E2-2 knockdown were identified. Among these genes, we confirmed that expression of Siglec-6 was inhibited by E2-2. Further more, Siglec-6 was expressed at a higher level on a human pDC subset with drastically lower expression of E2-2. Collectively, these results highlight that E2-2 modulates pDC function in a species-specific manner, which may provide insights for pDC development and functions.

Dendritic cells (DCs) are critical mediators for innate and adaptive immune responses. They are composed of two subsets: cDCs professional in antigen capture, processing and presentation, and pDCs dedicated to producing type I interferon (IFN-I) upon stimulation.

Although functional equivalences have been defined through comparative genomics^1^,^2^, there are notable discrepancies between human and mouse pDCs. Upon stimulation, mouse pDCs can produce IL-12 while human pDCs cannot^3^,^4^,^5^. Studies have suggested that, in response to LPS or CpG plus CD40L, human pDCs produce as much IL-12 as do cDCs but it’s subsequently proved to be resulted from contaminating cDCs^5^. The expression patterns of Toll like receptors by pDCs in mouse and human are different. Mouse pDCs express most TLRs except for TLR3^6^, while human pDCs only express TLR1, TLR6, and prominent TLR7 and TLR9^7^,^8^. Moreover, human and mouse pDCs express distinct surface markers. Human pDCs are marked by BDCA2, BDCA4, ILT7, CD123 and CD4. Of these markers, BDCA2 displays only 50.7% sequence identity at the amino acid level to its putative murine ortholog dectin-2^9^. Currently, anti-BDCA4 magnetic bead isolation kits are widely used to enrich human pDCs from blood or tissues, while mouse pDCs don’t express BDCA4 and they are enriched by anti-Bst2 magnetic beads. Mouse pDCs are marked by CD11c, Siglec-H, Bst2, B220, Gr-1, Ly-6C and Ly49Q. CD11c is a cDC marker both for human and mouse but human pDCs don’t express it. Mouse pDCs are uniquely characterized by highly expressed Ly49Q, a C-type lectin-like receptor, which is crucial for TLR9-mediated type I IFN and IL-12 production by mouse pDCs^10^,^11^. Besides, human pDCs express high levels of IL-3 receptor CD123 and are highly responsive to IL-3 stimulation^12^. However, Mouse pDCs express low levels of IL-3 receptor and do not respond to IL-3^13^.

E2-2 is preferentially expressed in human and mouse pDCs, and has been identified as a key transcription factor for pDC development and cell fate maintenance^14^,^15^. Constitutive or conditional deletion of E2-2 blocks the development of mouse pDCs but not other lineages of immune cells. The function of E2-2 in human pDCs has been explored via human hematopoietic stem cells (HSCs) differentiation assay *in vitro*. Over expression and RNAi-mediated knockdown of E2-2 suggest a critical role of it in pDC development^16^. In patients of Pitt-Hopkins Syndrome (PHS)^14^, which is associated with E2-2 haploinsufficiency, all major immune cell types are present in normal numbers in peripheral blood except for pDCs. In view of above differences between human and mouse pDCs, how E2-2 contributes to the species-specific differences is unclear yet.

In this report, we characterize the consequence of E2-2 reduction in a human pDC cell line GEN2.2. Function of E2-2 in human pDCs is in line with previous observation in mouse pDCs. Besides, cDNA array data indicates that E2-2 modulates the expression of some human specific genes including Siglec-6.

## Materials and Methods

### Cell Lines and Reagents

GEN2.2 cells were cultured with MS5 feeder cells in RPMI 1640 GlutaMAX (Invitrogen) supplemented with 10% (v/v) heat-inactivated fetal bovine serum (FBS, Invitrogen), 2 mM L-glutamine (Invitrogen), 100 μM non-essential amino acid (Invitrogen), 1 mM sodium pyruvate (Lab Amresco), 10 mM HEPES (Amresco), 100 units/ml penicillin and 100 μg/ml streptomycin (Invitrogen). HEK293T cells were grown in Dulbecco’s modified Eagle’s medium (DMEM) supplemented with 10% (v/v) heat-inactivated FBS (Hyclone), 2 mM L-glutamine, 10 mM HEPES, 100 units/ml penicillin and 100 μg/ml streptomycin. Mouse anti-HA (F-7) (sc-7392) antibody was from Santa Cruz Biotechnology. Rabbit polyclonal antibody to E2-2 (ab72586) was from Sigma. Rabbit polyclonal antibody against GFP was from Proteintech. Naïve CD4^+^ T cell Isolation Kit II (human) was from Miltenyi Biotec. 5(6)-Carboxyfluorescein diacetate N-succinimidyl ester (CFSE) was from Sigma. Quantitative PCR (qPCR) SYBR mix was from TIANGEN.

### Constructs, RNA Interference and Lentiviral Vectors Production

For knockdown experiments, the lentiviral vector FG12 described previously by Qin^17^ was used. Short hairpin shRNAs in the pLKO.1 lentiviral vector was purchased from Open Biosystem (Thermo Fisher Scientific, Pittsburgh PA, USA). The RNAi sequences specifically targeting E2-2 mRNA were as follows: E2-2i 1#, 5’-GAAAGGAATCTGAATCCGAAA-3’; E2-2i 2#, 5’-CACGAAATCTTCGGAGGACAA-3’. The U6 RNAi sequence cassette was then subcloned into FG12. An RNAi construct with only U6 promoter was used as control. Vectors were harvested at 48 h post transfection of 293T cells with lentiviral vector, Gag-Pol (ΔNRF) and Env (Vsvg). GEN2.2 cells were transduced with lentiviral vectors for 3 h and polybrene (4 μg/ml) added.

### RT- PCR and ELISA

For E2-2 knockdown, GEN2.2 cells were transduced by lentiviral vectors with or without shRNA for E2-2, and GFP^+^ cells were sorted at 3–5 days post infection. Cells were resuspended in Trizol reagent (Invitrogen), total RNA was isolated according to Molecular Cloning: A Laboratory Manual and reverse transcribed using a poly-dT oligonucleotide (Invitrogen) and M-MLV Reverse Transcriptase (Promega) at 42 °C for 1 hr. PCR assays were carried out with Corbett 6200/6600 qPCR System using 5 μl cDNA template, 0.25 μM of each primer and Taq polymerase in 1 × SYBR Green Mix. Reaction conditions were as follows: a 10 min pre-denaturation step at 95 °C was followed by 40 cycles of 10 s at 95 °C, 15 s at 55 °C, and 15–30 s at 72 °C. Primers used for the measurement of indicated gene expression were as follows: E2-2, 5’-GAGTGTCTCCTCTGGCAGC-3’ and 5’-CCATGTGATTCGATGCGTC-3’; CLEC4C (BDCA2), 5’-ACTGGGATGCAATCTTGGAC-3’ and 5’-GATCTGACAGCCCCAGAAAA-3’; Siglec-6, 5’- AAGGGGCTGATGTTCCAGTG-3’ and 5’- ATGCAGCATTGTCCCTCCTC-3’; TCL1B, 5’-TTCCAGTTTCTGGGAAATAGCAG-3’ and 5’-TCTCCGGCTGATATGTTAGGAC-3’; GZMB, 5’-TACCATTGAGTTGTGCGTGGG-3’ and 5’-GCCATTGTTTCGTCCATAGGAGA-3’. Quantitation was normalized to an endogenous EF1α control. For an ELISA, infected GEN2.2 cells were cultured in the presence of different concentrations of type B CpG (ODN2006, Invitrogen) for 20 hr. Culture supernatants were collected and analyzed by human-specific IFNα and IL-6 ELISA kit (Bender MedSystems).

### Western Blot Analysis

Cells were lysed at 48 h after transfection of E2-2 expression plasmid and control shRNA or E2-2-specific shRNA using PEI (Sigma). Samples were separated by SDS-PAGE and transferred to PVDF membrane. After blocking in TBS containing 5% skim-milk, the blots were probed with indicated antibodies.

### Flow Cytometry

Monoclonal antibodies to CD3, CD11c, CD14, CD16, CD20, CD123, and HLA-DR conjugated to FITC, APC-Cy7, APC, PerCP_Cy5.5 were purchased from BioLegend, and Live/Dead Fixable Yellow Dead Cell Stain Kit from Invitrogen. Mouse monoclonal antibody to human Siglec-6 was prepared in mouse according to Current Protocols in Immunology. Single cell suspensions were stained and analyzed with FACS Fortessa (BD). Cell sorting was performed with FACS Aria III sorter (BD) and data were analyzed with Summit software (Dako Cytomation).

### Isolation of Human Primary pDCs

Human PBMCs were stained with mouse antibody mix to human CD3, CD14, CD16, and CD19. Lineage-positive cells were depleted by goat anti-mouse IgG (H + L) microbeads with MACS Columns and MACS Separators (Miltenyi Biotec). The negative cells were stained with CD123 and HLA-DR. CD123^+^HLA-DR^+^ cells were sorted by FACS (>95% purity).

### T cells Proliferation

GEN2.2 cells transduced with lentiviral vector expressing shRNAs of E2-2 at 3–5 days post infection were sorted by FACS Aria III sorter (BD). Naive CD4^+^CD45RA^+^ T lymphocytes (>95% purity) were isolated from human PBMC via negative immunomagnetic depletion (Miltenyi Biotech), labeled with 5 μM CFSE for 10 min at room temperature and quenched by adding an equal volume of FBS. GEN2.2 and T cell mix was cultured in a 96-well round-bottom plate. 7 days later, cells were harvested and analyzed by flow cytometry.

### Gene Expression Profiling

For microarray analysis, E2-2 shRNA transduced GEN2.2 cells were sorted and resuspended in Trizol reagent (Invitrogen), and total RNA was reverse transcribed, amplified with a MessageAmp^TM^ Premier RNA Amplification Kit (Ambion) and labeled, fragmented and hybridized to Human Genome U133 Plus 2.0 arrays (Affymetrix). Chips were analyzed using a Laser Scan Confocol microarray reader (CapitalBio, Beijing, China). Raw data were transformed with the RMA algorithm, which yields a normalized expression value, and “absent” and “present” calls. Normal gene expression profiles of human blood leukocytes were derived from supplementary materials and EBI Array Express data set E-TABM-34^1^.

### Statistical Analysis

Statistical significance was determined by unpaired, two-tailed Student’s t test. *P* value < 0.05 was considered statistically significant. Data were analyzed with GraphPad Prism software version 5.0 (GraphPad Software). The methods used for analysis of microarray data were described below.

## Results

### Short hairpin RNAs knock down E2-2 in GEN2.2 cells

**Figure 1:**
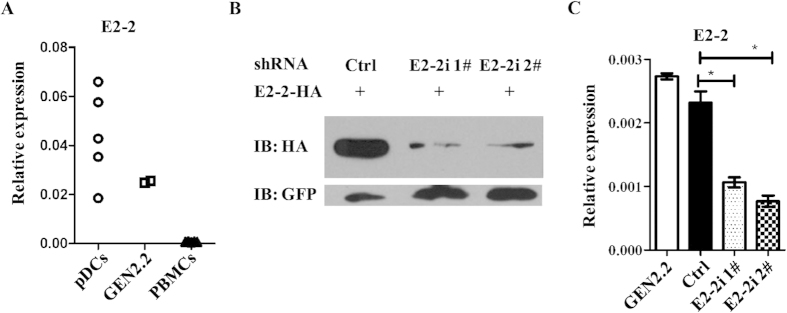
E2-2 expression in GEN2.2 is silenced by shRNAs. (**A**) E2-2 expression in GEN2.2 cells, purified human pDCs and PBMCs (n = 5) was measured by real-time PCR. (**B**) Immunobloting analysis of E2-2 in lysates of 293T cells transfected with E2-2 overexpression plasmid and control shRNA or E2-2-specific shRNA. GFP was used as a loading control. (**C**) E2-2 expression in GEN2.2 cells transduced with or without control shRNA or E2-2-specific shRNA was measured by real-time PCR.. * indicated p < 0.05. All the gels were run under the same experimental conditions as detailed in the Methods section, and full-length blots were cropped for final display.

The GEN2.2 cells resemble primary human pDCs with similar phenotype and function^18^,^19^. E2-2 was expressed abundantly in human pDCs, and at similar levels in GEN2.2 cells (Fig. 1A). We selected four short hairpin RNAs (shRNAs) for E2-2, all of which could knock down E2-2 expression in transfected 293T cells (Figure S1). Of the four shRNAs, two could knockdown E2-2 expression efficiently (Fig. 1B and Figure S2). These two shRNAs were chosen for construction of lentiviral vectors for E2-2 knockdown in GEN2.2 cells. The lentiviral vector carries independent GFP expression cassette, which is a reporter and selection marker for cells successfully transduced by E2-2 shRNA. GEN2.2 cells can hardly be transfected with plasmid and lentiviral vector enables efficient gene transfer in the absence of cell phenotypic and functional maturation^20^.

Quantitative RT-PCR (qRT-PCR) with GFP^+^GEN2.2 cells showed that more than 50% of E2-2 transcription was significantly suppressed (Fig. 1C). Comparable E2-2 expression was observed in GEN2.2 cells transduced with control lentiviral vector, which indicated that infection didn’t affect E2-2 expression.

### E2-2 silence in GEN2.2 cells abrogates IFN-α production but induces proliferation of naïve T lymphocytes.

**Figure 2:**
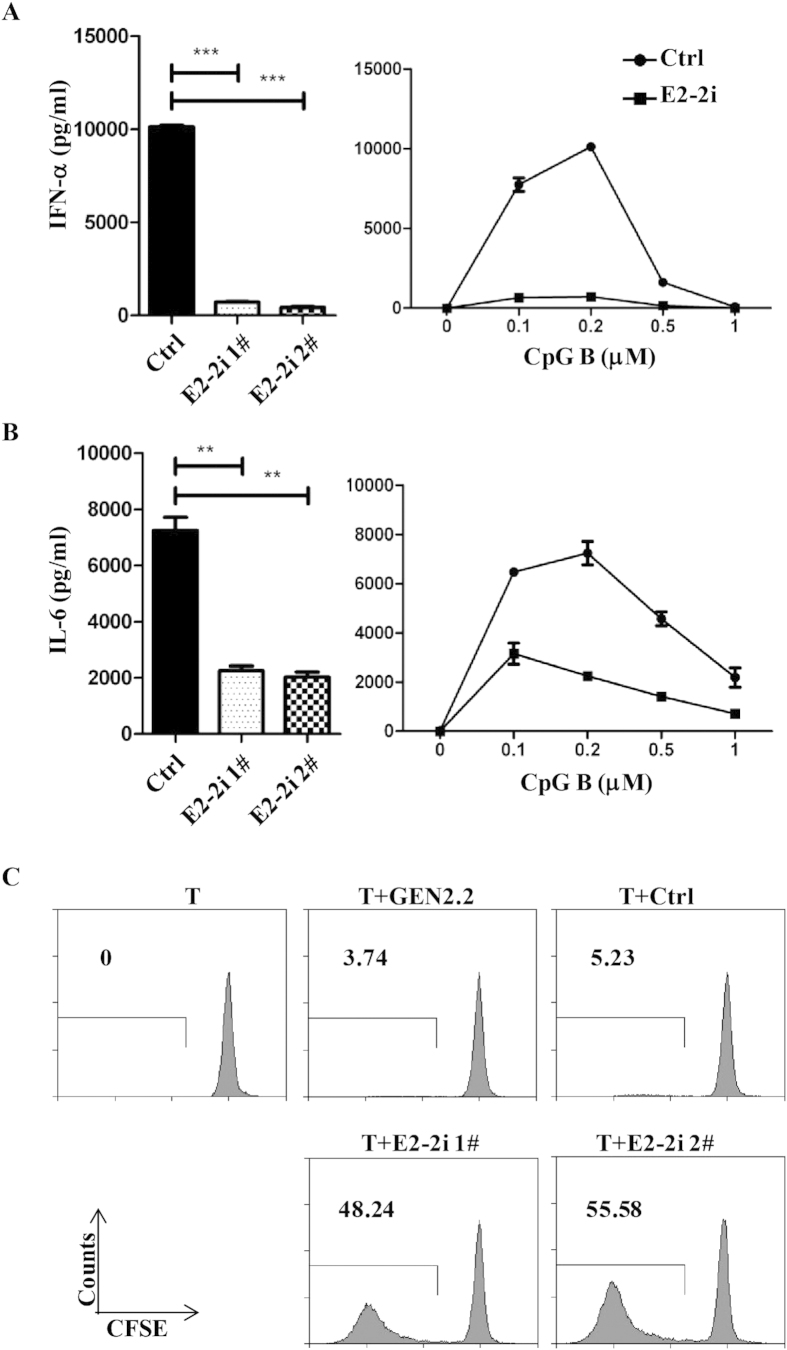
Decreased IFNα and IL-6 production and higher T cell proliferation by E2-2 knockdown GEN2.2 cells. (**A**) and (**B**) GEN2.2 cells were incubated with CpG B for 20 h. Different concentrations (0 ~ 1 μM) of CpG B stimulated cell supernantant was analyzed (right panel) and 0.2 μM was shown (left panel). (**A**) IFN-α in supernatants of E2-2 knockdown and control GEN2.2 cells was measured by ELISA. *** indicated *p* < 0.001. (**B**) IL-6 in supernatants of E2-2 knockdown and control GEN2.2 cells was measured by ELISA. ** indicated *p* < 0.01. (**C**) T cell priming capacity of E2-2 knockdown GEN2.2 cells. GEN2.2 cells transduced with or without control shRNA or E2-2-specific shRNA were co-cultured with CD4^+^CD45RA^+^ T cells, which were purified and labeled with CFSE. 7 days later, cells were analyzed for CFSE levels by flow cytometry. Numbers indicated percentage of CFSE^low^(proliferated) cells. Data are representative of at least three independent experiments.

GEN2.2 cells produce IFN-α in response to unmethylated DNA (CpG). IFN-α secretion after CpG B (0 ~ 1 μM) stimulation was detected in control GEN2.2 cells and peaked at 0.2 μM. However, E2-2 knockdown GEN2.2 cells could barely secret any IFN-α in response to CpG B (Fig. 2A). Additionally, the level of inflammatory cytokine IL-6 was reduced to 30% of control cells (Fig. 2B). Thus, E2-2 was indispensable for pDCs to yield tremendous IFN-α upon stimulation.

After 48h activation with IL-3 plus CD40L, with virus or with the three signals, matured GEN2.2 cells can stimulate naive T lymphocyte proliferation^18^. Without activation, GEN2.2 cells show poor capacity to prime T cells. We co-cultured the GEN2.2 cells with freshly isolated naïve CD4^+^ T cells, which were pre-labeled with CFSE. GEN2.2 cells and control lentiviral vector transduced cells could barely induce T cell proliferation (~3%). However, without any stimulation, ~50% T cells proliferated once co-cultured with E2-2 knockdown GEN2.2 cells (Fig. 2C). FACS analysis of costimulatory molecules showed increased expression of CD40, CD83 and CD86 after E2-2 down-regulation (Figure S3). Thus, we concluded that GEN2.2 cells acquired antigen presentation capacity after E2-2 knockdown.

### E2-2 reduction in GEN2.2 cells down-regulates pDC signature genes and up-regulates cDC signature genes

**Figure 3:**
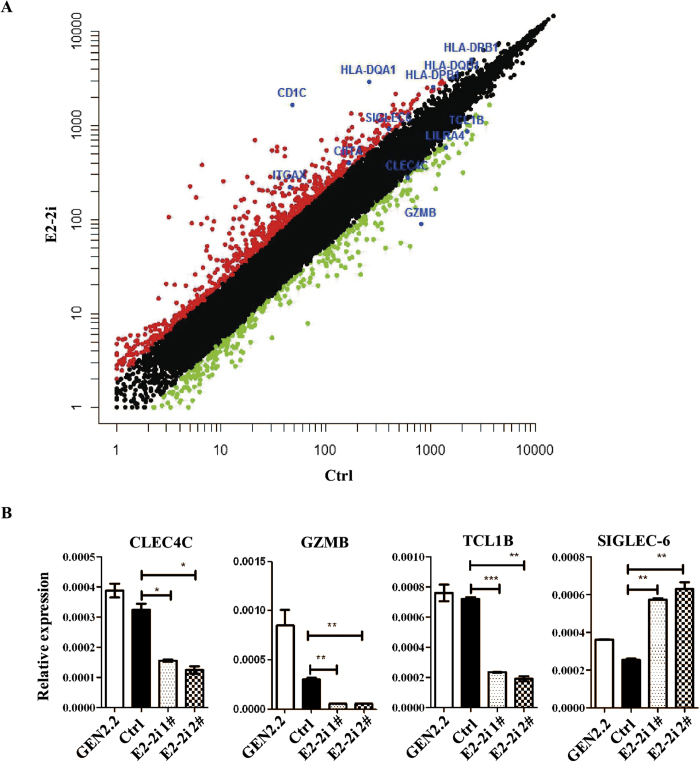
Gene program regulated by E2-2 in human pDCs. (**A**) Pairwise comparison of expression profile for E2-2 knockdown and control GEN2.2 cells. The scatter plot represents normalized log intensities of individual probes, with the probes increased or decreased >2 fold in GEN2.2 cells with E2-2-specific shRNA (E2-2i) and control shRNA (Ctrl) indicated in red and green, respectively. Some probes are highlighted in blue. (**B**) Expression of CLEC4C, GZMB, TCL1B and Siglec-6 in GEN2.2 cells with or without control shRNA or E2-2-specific shRNA. *, ** and *** indicated p < 0.05, p < 0.01 and p < 0.001.

E2-2 controls the gene expression program of mouse pDCs. As a transcription factor, E2-2 can directly bind the regulatory regions of some pDC specific genes^14^. To identify gene program regulated by E2-2 in human pDCs, E2-2 knockdown GEN2.2 cells were analyzed by expression microarrays. E2-2 expression was downregulated to about 50% as compared with control cells by all eight probesets. Expression of housekeeping genes (*GAPDH* and *Actin*) were at the same level in E2-2 knockdown and control GEN2.2 cells (data not shown). Significance analysis of microarray (SAM) showed that 827 probe sets were differentially expressed in E2-2 knockdown and control GEN2.2 cells by more than two-folds: 628 probe sets were expressed at higher abundance in E2-2 knockdown GEN2.2 cells as compared with control GEN2.2 cells. And 199 probe sets were expressed at lower abundance in E2-2 knockdown GEN2.2 cells as compared with control GEN2.2 cells (Fig. 3A).

To exclude those leukemia-related genes^21^ and observe the change of DC specific gene expression program, we constructed a list of “DC signature” probe sets which were defined by highest expression in pDCs or cDCs via evaluation of overall gene expression in human blood leukocytes with Wilcoxon signed-rank test (P < 0.05). Gene expression profiles of monocytes, neutrophils, pDCs, BDCA1^+^cDCs, BDCA3^+^cDCs, CD8^+^T, CD4^+^T, NK and B cells were retrieved from public databases of previous publication^1^.

Then the overlap of these “DC signature” genes and differentially expressed genes in E2-2 knockdown GEN2.2 cells showed that all cDC signature sets (17/17) were up-regulated while most pDC signature sets (15/18) were down-regulated (Table 1). Human pDC surface markers like *CLEC4C* (*BDCA2*)(Fig. 3B), *LILRA4* (*ILT7*) were included in the down-regulated set, while cDC highly expressed class II transactivator (*CIITA*), which is essential for *MHCII* expression, was present in the up-regulated set (Fig. 3A). In accordance with that, expression levels of *HLA-DQA1*, *HLA-DRB1*, *HLA-DQB1* and *HLA-DPB1* were increased (Fig. 3A and Table 1).

Among the pDC signature genes, GZMB was the most down-regulated gene (E2-2i/Ctrl ratio = 0.11, Fig. 3B). Granzymes such as GZMB represent a major component of the granules of cytotoxic cells, like cytotoxic T lymphocytes (CTLs) and NK cells. CTLs recognize target cells and secrets granules, which will induce cell apoptosis. Recent report suggested that human pDCs were also an abundant source of enzymatically active GZMB protein and they may produce GZMB in amounts that exceed GZMB produced by classical cytotoxic lymphocytes^22^. These pDC-derived GZMB suppresses T cell proliferation in perforin-independent manner. Down-regulated GZMB in E2-2 knockdown GEN2.2 cells may result in higher proliferation of T cells. For mouse, GZMB could only be detected in NK cells and mast cells but not pDCs.

### Human specific genes regulated by E2-2

To define species-specific features of human pDCs regulated by E2-2, we submitted above differentially expressed genes to the Database for Annotation, Visualization and Integrated Discovery (DAVID) and analyzed them with functional annotation tool^23^. A gene list encompassing thirty genes which were expressed in human but not mouse was produced via limiting annotations by homo sapiens or mus musculus and aligning the two groups (Table 2). We found some well characterized genes (such as *CLEC4C* and *LILRA4*) as well as several novel genes not reported previously.

Toll-like receptors play an essential role in innate immunity against pathogens. pDCs recognize DNA virus and CpG or single-stranded viral RNA for their selectively expression of TLR7 and TLR9. Associated with MyD88, TLR10 is weakly expressed in human pDCs^8^ and preferentially expressed on CD1a^+^ subset of cDCs^24^. Expression of TLR10 has not been detected in mouse^25^. Human TLR10 is an anti-inflammatory pattern-recogenition receptor without known ligand^24^. Once E2-2 was down-regulated, GEN2.2 promoted cDC signature genes expression and TLR10 would be one of them.

TCL1B is a protooncogene and shares 60% sequence homology with the other family member TCL1A. Intracellular TCL1A expression is considered to be a marker for plasmacytoid dendritic cell leukemia. About 37% blood primary pDCs express TCL1A, however, it’s highly expressed in most pDC leukemia samples^26^. Intracellular staining showed that all GEN2.2 cells express TCL1A (Figure S4). Our results showed that TCL1B was down-regulated after E2-2 silence (Fig. 3B).

**Figure 4:**
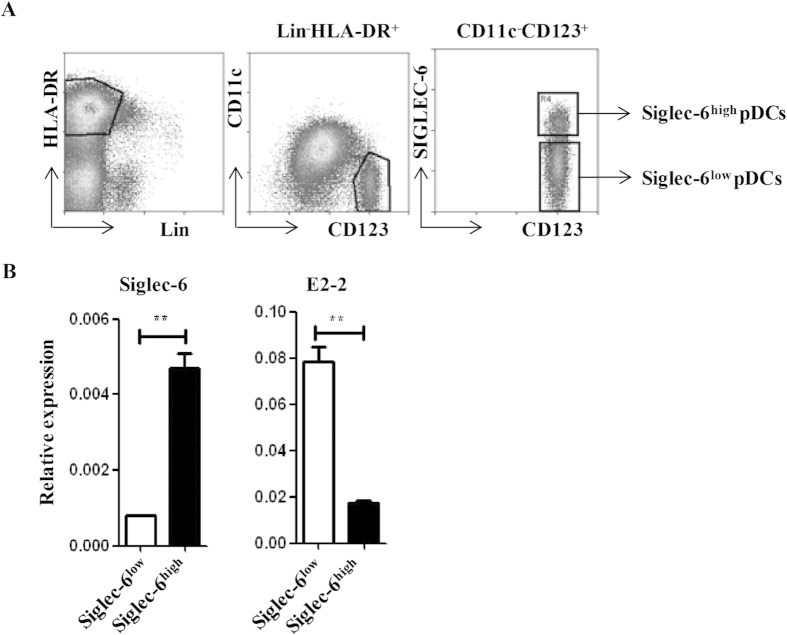
Siglec-6 and E2-2 expression on human pDC subsets. (**A**) Flow cytometry analysis of Siglec-6 expression on human pDCs from lineage negative PBMCs. Primary pDCs were marked as HLA-DR^-^CD11c^-^CD123^+^ and divided into two subsets according to Siglec-6 expression. (**B**) Siglec-6 and E2-2 expression in Siglec-6^high^ or Siglec-6^low^ pDC subsets. ** indicated p < 0.01. Data are representative of at least three donors.

Sialic acid-binding immunoglobulin-like lectin 6 (Siglec-6) is mainly expressed on immune cells and placental trophoblast^27^,^28^. As an inhibitory receptor, Siglec-6 has a dominant effect on both the proliferation and effector functions of tissue-like memory B cells^29^. It has been reported that Siglec-6 is expressed on plasmacytoid dendritic cell leukemia but not cutaneous myeolomonocytic leukemia (c-AML)^30^. qRT-PCR result confirmed augmented expression of Siglec-6 in GEN2.2 cells after E2-2 knockdown (Fig. 3B). To test Siglec-6 expression on human primary pDCs, we prepared monoclonal antibody against human Siglec-6, and the antibody was conjugated with PE (CD327-PE). According to Siglec-6 expression, human pDCs in PBMCs were divided into two subsets: Siglec-6^high^ pDCs and Siglec-6^low^ pDCs (Fig. 4A). Different Siglec-6 expression at mRNA level was validated by qRT-PCR. Siglec-6^low^ pDCs express ~4 folds higher E2-2 than Siglec-6^high^ pDCs (Fig. 4B). Besides, there were three E2-2 binding motifs (CANNTG) 500 bp upstream ATG of Siglec-6 coding region, which were potential binding sites for E2-2 (data not shown). Thus, we propose that E2-2 inhibits Siglec-6 expression in pDCs.

Previously a ChIP-on-chip array was performed in human pDC cell line CAL-1. A high-confidence list of 2,477 genes was defined as E2-2 binding targets^15^. The overlap of these targets and the differentially expressed genes comprised 90 genes, which were bound to and regulated directly by E2-2 (Table S1). Other than well known pDC and cDC specific genes, we identified genes that were not yet known to be involved in the biology of pDCs. Take SPTDN1 for example, recent report showed that it was an important host target for IL-27 to inhibit HIV-1 infection in human macrophages^31^. pDCs could be infected by HIV and whether SPTBN1 was included was still unknown. Among these genes bound to and regulated directly by E2-2, *C20orf26*, *CD1E*, *FCRL4*, *LILRA4*, *TCL1B* and *TP53I3* were human specific genes. FCRL4 was one of the most up-regulated genes (E2-2i/Ctrl ratio = 17.77) in E2-2 knockdown GEN2.2 cells. As an inhibitory receptor, FCRL4 contributes to HIV-associated human B cell exhaustion^29^, and is considered as a molecular switch in B cells to dampen adaptive immune signaling and enhance innate signaling in response to chronic antigenic stimulation^32^. pDCs has been described as the bridge for innate and adaptive immunity^33^. Whether the function of FCRL4 on pDCs as a molecular switch is inversed has never been reported. Thus, we have defined novel specific genes regulated by E2-2 which can be candidates for further investigation in human pDCs.

## Discussion

Here we investigated the function of E2-2 in human pDC functions. Consistent with that in mouse, loss of E2-2 in human pDCs eliminated IFN response to unmethylated DNA, inhibited pDC signature genes expression and elevated antigen presentation ability without any stimulation.

Heterogeneity of pDCs has been proposed ever since 2005. Kamogawa-Schifter provides the first evidence that murine pDCs can be divided into two subsets according to the expression of Ly49Q^34^. Ly49Q^−^ pDCs are procursors of Ly49Q^+^ pDCs. RAG1, Ig rearrangement products^35^ and CD4^36^ are also used to demonstrate that murine pDCs constitute a heterogeneous cell population. CCR9 has been used to distinguish functionally distinct pDC subsets but finally it proves that B220^+^CCR9^−^ DCs are not pDCs but precursors of cDCs^37^,^38^. For human pDCs, CD2 contributes to define pDC heterogeneity^39^. Here we identify two pDC subsets: Siglec-6^high^ and Siglec-6^low^. Both of them were Lin-HLA-DR+CD11c-CD123+ and express the transcription factor E2-2. Siglec-6^high^ pDCs express lower E2-2 and their developmental pathway should be well studied to ascertain whether they are pDC precursor or cDC precursor or another cell subset.

Most Siglecs have two conserved intracellular domains: ITIM and ITIM-like motif, capable of inhibiting signaling. For example, mouse eosinophils and activated T cells express Siglec-F, which is a functional counterpart of human Siglec-8 and controls responses of eosinophils and helper T cells^40^,^41^. Loss of Siglec-F will increase bone-marrow, blood and tissue eosinophilia in a model of induced lung allergy^42^. In HIV-associated B cell exhaustion, Siglec-6 is reported to inhibit BCR-induced proliferation and effector function of tissue-like memory B cells^29^. The role of Siglec-6 in human pDCs has never been investigated. Recent report show extensive and direct TLR-Siglec interactions negatively regulate the function of multiple TLRs. Among them, Siglec-6 weakly interacted with TLR9 and strongly interacted with TLR10 expressed from human THP-1^43^. After E2-2 knockdown, Siglec-6 and TLR10 were up-regulated and their strong interaction may inhibit pDC response to TLR ligand (CpG B) and explain abrogated IFN-α production.

Importantly, our study confirms the function of E2-2 in human pDCs and reveals a number of human genes regulated by E2-2, delineating a non-redundant role of E2-2 in refining species gap between human and mouse pDCs.

## Additional Information

**How to cite this article**: Cheng, M. et al. Characterization of species-specific genes regulated by E2-2 in human plasmacytoid dendritic cells. *Sci. Rep.* **5**, 10752; doi: 10.1038/srep10752 (2015).

## Supplementary Material

Supplementary Information

Supplementary Table S1

## Figures and Tables

**Table 1:** Differentially expressed DC signature genes in E2-2 knockdown GEN2.2 cells.

**Gene symbol**	**Gene title**	**Ratio**
**cDC signature genes**		
LY86	lymphocyte antigen 86	7.6826
**HLA-DQA1**	major histocompatibility complex, class II, DQ alpha 1	4.7388
EMP1	epithelial membrane protein 1	3.6011
**HLA-DQB1**	major histocompatibility complex, class II, DQ beta 1	2.6273
PSEN2	presenilin 2 (Alzheimer disease 4)	2.533
**HLA-DPB1**	major histocompatibility complex, class II, DP beta 1	2.4325
DISC1	disrupted in schizophrenia 1	2.4288
**CIITA**	class II, major histocompatibility complex, transactivator	2.4064
CSRP1	cysteine and glycine-rich protein 1	2.2192
PPM1H	protein phosphatase 1H (PP2C domain containing)	2.2087
KCNMB1	potassium large conductance calcium-activated channel, subfamily M, beta member 1	2.2002
ENTPD1	ectonucleoside triphosphate diphosphohydrolase 1	2.1907
CENTA2	ArfGAP with dual PH domains 2	2.1818
IER5	immediate early response 5	2.1753
NAB2	NGFI-A binding protein 2 (EGR1 binding protein 2)	2.1598
SPRY2	sprouty homolog 2 (Drosophila)	2.0886
ATP1B1	ATPase, Na+/K+ transporting, beta 1 polypeptide	2.0514
**pDC signature genes**		
**GZMB**	granzyme B	0.1093
RGS7	regulator of G-protein signaling 7	0.2939
COBLL1	COBL-like 1	0.3393
SRPX	sushi-repeat-containing protein, X-linked	0.3716
**TCL1B**	T-cell leukemia/lymphoma 6	0.3829
**LILRA4**	leukocyte immunoglobulin-like receptor, subfamily A (with TM domain), member 4	0.417
MAP1A	microtubule-associated protein 1A	0.4371
DUSP5	dual specificity phosphatase 5	0.4389
MYB	v-myb myeloblastosis viral oncogene homolog (avian)	0.4568
**CLEC4C**	C-type lectin domain family 4, member C	0.4609
MLF1IP	MLF1 interacting protein	0.461
SLC12A3	solute carrier family 12, member 3	0.4707
RRBP1	transforming growth factor, beta-induced, 68kDa	0.4711
TGFBI	ribosome binding protein 1 homolog 180kDa (dog)	0.4711
KCNK1	potassium channel, subfamily K, member 1	0.4809
ENPP2	ectonucleotide pyrophosphatase/phosphodiesterase 2	2.0998
RASGEF1B	RasGEF domain family, member 1B	2.4254
DAB2	disabled homolog 2, mitogen-responsive phosphoprotein (Drosophila)	3.4648

**Table 2:** Differentially expressed human specific genes in E2-2 knockdown GEN2.2 cells.

**Gene Symbol**	**Gene Title**	**Ratio**
**HLA-DQA1**	major histocompatibility complex, class II, DQ alpha 1	4.7388
**HLA-DRB1**	major histocompatibility complex, class II, DR beta 1	3.6552
C11orf21	chromosome 11 open reading frame 21	3.6179
CA2	carbonic anhydrase II	3.1831
ANKH	ankylosis, progressive homolog (mouse)	3.0989
**HLA-DQB1**	major histocompatibility complex, class II, DQ beta 1	2.7546
LOC100288152	hypothetical protein LOC100288152	2.7061
**TLR10**	toll-like receptor 10	2.687
HIST1H2BD	histone cluster 1, H2bd	2.5222
HIST1H2BC	histone cluster 1, H2bc	2.4774
HLA-DPB1	major histocompatibility complex, class II, DP beta 1	2.4325
**Siglec-6**	sialic acid binding Ig-like lectin 6	2.3482
H2BFS	H2B histone family, member S	2.2932
ZNF827	zinc finger protein 827	2.2415
CLEC2B	C-type lectin domain family 2, member B	2.2331
SP140L	SP140 nuclear body protein-like	2.1677
C1orf228	chromosome 1 open reading frame 228	2.0496
**SPTBN1**	spectrin, beta, non-erythrocytic 1	0.4835
SERHL2	serine hydrolase-like 2	0.4626
**CLEC4C**	C-type lectin domain family 4, member C	0.4609
LOC150568	hypothetical LOC150568	0.4609
MAP1A	microtubule-associated protein 1A	0.4371
**LILRA4**	“leukocyte immunoglobulin-like receptor, subfamily A (with TM domain), member 4”	0.417
FLJ35024	hypothetical LOC401491	0.4129
IGHA1	immunoglobulin heavy constant alpha 1	0.4067
IGHM	immunoglobulin heavy constant mu	0.3864
**TCL1B**	T-cell leukemia/lymphoma 1B	0.3829
IGLC1	Immunoglobulin lambda constant 1 (Mcg marker)	0.3147
ZNF385B	zinc finger protein 385B	0.234
MAP1B	microtubule-associated protein 1B	0.1995

## References

[b1] Robbins S. H. *et al.* Novel insights into the relationships between dendritic cell subsets in human and mouse revealed by genome-wide expression profiling. Genome Biol. 9, R17 (2008). 18218067 10.1186/gb-2008-9-1-r17 PMC2395256

[b2] Crozat K. *et al.* Comparative genomics as a tool to reveal functional equivalences between human and mouse dendritic cell subsets. Immunol. Rev. 234, 177–198 (2010). 20193019 10.1111/j.0105-2896.2009.00868.x

[b3] Asselin-Paturel C. *et al.* Mouse type I IFN-producing cells are immature APCs with plasmacytoid morphology. Nat. Immunol. 2, 1144–1150 (2001). 11713464 10.1038/ni736

[b4] Bjorck P. Isolation and characterization of plasmacytoid dendritic cells from Flt3 ligand and granulocyte-macrophage colony-stimulating factor-treated mice. Blood 98, 3520–3526 (2001). 11739152 10.1182/blood.v98.13.3520

[b5] Ito T., Kanzler H., Duramad O., Cao W. & Liu Y. J. Specialization, kinetics, and repertoire of type 1 interferon responses by human plasmacytoid predendritic cells. Blood 107, 2423–2431 (2006). 16293610 10.1182/blood-2005-07-2709

[b6] Edwards A. D. *et al.* Toll-like receptor expression in murine DC subsets: lack of TLR7 expression by CD8 alpha^+^ DC correlates with unresponsiveness to imidazoquinolines. Eur. J. Immunol. 33, 827–833 (2003). 12672047 10.1002/eji.200323797

[b7] Krug A. *et al.* Toll-like receptor expression reveals CpG DNA as a unique microbial stimulus for plasmacytoid dendritic cells which synergizes with CD40 ligand to induce high amounts of IL-12. Eur. J. Immunol. 31, 3026–3037 (2001). 11592079 10.1002/1521-4141(2001010)31:10<3026::aid-immu3026>3.0.co;2-h

[b8] Hornung V. *et al.* Quantitative expression of toll-like receptor 1-10 mRNA in cellular subsets of human peripheral blood mononuclear cells and sensitivity to CpG oligodeoxynucleotides. J. Immunol. 168, 4531–4537 (2002). 11970999 10.4049/jimmunol.168.9.4531

[b9] Dzionek A. *et al.* BDCA-2, a novel plasmacytoid dendritic cell-specific type II C-type lectin, mediates antigen capture and is a potent inhibitor of interferon alpha/beta induction. J. Exp. Med. 194, 1823–1834 (2001). 11748283 10.1084/jem.194.12.1823 PMC2193584

[b10] Rahim M. M. *et al.* Ly49Q positively regulates type I IFN production by plasmacytoid dendritic cells in an immunoreceptor tyrosine-based inhibitory motif-dependent manner. J. Immunol. 190, 3994–4004 (2013). 23479228 10.4049/jimmunol.1200873

[b11] Yoshizaki M. *et al.* Spatiotemporal regulation of intracellular trafficking of Toll-like receptor 9 by an inhibitory receptor, Ly49Q. Blood 114, 1518–1527 (2009). 19528537 10.1182/blood-2008-12-192344

[b12] Grouard G. *et al.* The enigmatic plasmacytoid T cells develop into dendritic cells with interleukin (IL)-3 and CD40-ligand. J. Exp. Med. 185, 1101–1111 (1997). 9091583 10.1084/jem.185.6.1101 PMC2196227

[b13] O’Keeffe M. *et al.* Mouse plasmacytoid cells: long-lived cells, heterogeneous in surface phenotype and function, that differentiate into CD8(+) dendritic cells only after microbial stimulus. J. Exp. Med. 196, 1307–1319 (2002). 12438422 10.1084/jem.20021031 PMC2193989

[b14] Cisse B. *et al.* Transcription factor E2-2 is an essential and specific regulator of plasmacytoid dendritic cell development. Cell 135, 37–48 (2008). 18854153 10.1016/j.cell.2008.09.016 PMC2631034

[b15] Ghosh H. S., Cisse B., Bunin A., Lewis K. L. & Reizis B. Continuous expression of the transcription factor e2-2 maintains the cell fate of mature plasmacytoid dendritic cells. Immunity 33, 905–916 (2010). 21145760 10.1016/j.immuni.2010.11.023 PMC3010277

[b16] Nagasawa M., Schmidlin H., Hazekamp M. G., Schotte R. & Blom B. Development of human plasmacytoid dendritic cells depends on the combined action of the basic helix-loop-helix factor E2-2 and the Ets factor Spi-B. Eur. J. Immunol. 38, 2389–2400 (2008). 18792017 10.1002/eji.200838470

[b17] Qin X. F., An D. S., Chen I. S. & Baltimore D. Inhibiting HIV-1 infection in human T cells by lentiviral-mediated delivery of small interfering RNA against CCR5. Proc. Natl. Acad. Sci. USA 100, 183–188 (2003). 12518064 10.1073/pnas.232688199 PMC140921

[b18] Chaperot L. *et al.* Identification of a leukemic counterpart of the plasmacytoid dendritic cells. Blood 97, 3210–3217 (2001). 11342451 10.1182/blood.v97.10.3210

[b19] Chaperot L. *et al.* Leukemic plasmacytoid dendritic cells share phenotypic and functional features with their normal counterparts. Eur. J. Immunol. 34, 418–426 (2004). 14768046 10.1002/eji.200324531

[b20] Veron P. *et al.* Highly efficient transduction of human plasmacytoid dendritic cells without phenotypic and functional maturation. J. Transl. Med. 7, 10 (2009). 19173717 10.1186/1479-5876-7-10 PMC2657113

[b21] Sapienza M. R. *et al.* Molecular profiling of blastic plasmacytoid dendritic cell neoplasm reveals a unique pattern and suggests selective sensitivity to NF-kB pathway inhibition. Leukemia 28, 1606–1616 (2014). 24504027 10.1038/leu.2014.64 PMC4294271

[b22] Jahrsdorfer B. *et al.* Granzyme B produced by human plasmacytoid dendritic cells suppresses T-cell expansion. Blood 115, 1156–1165 (2010). 19965634 10.1182/blood-2009-07-235382 PMC2920226

[b23] Huang da W., Sherman B. T. & Lempicki R. A. Systematic and integrative analysis of large gene lists using DAVID bioinformatics resources. Nat. Protoc. 4, 44–57 (2009). 19131956 10.1038/nprot.2008.211

[b24] Oosting M. *et al.* Human TLR10 is an anti-inflammatory pattern-recognition receptor. Proc. Natl. Acad. Sci. U.S.A. 111, E4478–4484 (2014). 25288745 10.1073/pnas.1410293111 PMC4210319

[b25] Hasan U. *et al.* Human TLR10 is a functional receptor, expressed by B cells and plasmacytoid dendritic cells, which activates gene transcription through MyD88. J. Immunol. 174, 2942–2950 (2005). 15728506 10.4049/jimmunol.174.5.2942

[b26] Angelot-Delettre F. *et al.* Intracytoplasmic detection of TCL1--but not ILT7-by flow cytometry is useful for blastic plasmacytoid dendritic cell leukemia diagnosis. Cytometry A. 81, 718–724 (2012). 22674796 10.1002/cyto.a.22072

[b27] Patel N. *et al.* OB-BP1/Siglec-6. a leptin- and sialic acid-binding protein of the immunoglobulin superfamily. J. Biol. Chem. 274, 22729–22738 (1999). 10428856 10.1074/jbc.274.32.22729

[b28] Brinkman-Van der Linden E. C. *et al.* Human-specific expression of Siglec-6 in the placenta. Glycobiology 17, 922–931 (2007). 17580316 10.1093/glycob/cwm065

[b29] Kardava L. *et al.* Attenuation of HIV-associated human B cell exhaustion by siRNA downregulation of inhibitory receptors. J. Clin. Invest. 121, 2614–2624 (2011). 21633172 10.1172/JCI45685 PMC3127436

[b30] Dijkman R. *et al.* Gene-expression profiling and array-based CGH classify CD4+CD56+ hematodermic neoplasm and cutaneous myelomonocytic leukemia as distinct disease entities. Blood 109, 1720–1727 (2007). 17068154 10.1182/blood-2006-04-018143

[b31] Dai L. *et al.* IL-27 inhibits HIV-1 infection in human macrophages by down-regulating host factor SPTBN1 during monocyte to macrophage differentiation. J. Exp. Med. 210, 517–534 (2013). 23460728 10.1084/jem.20120572 PMC3600911

[b32] Sohn H. W., Krueger P. D., Davis R. S. & Pierce S. K. FcRL4 acts as an adaptive to innate molecular switch dampening BCR signaling and enhancing TLR signaling. Blood 118, 6332–6341 (2011). 21908428 10.1182/blood-2011-05-353102 PMC3236118

[b33] Liu Y. J. IPC: professional type 1 interferon-producing cells and plasmacytoid dendritic cell precursors. Annu. Rev. Immunol. 23, 275–306 (2005). 15771572 10.1146/annurev.immunol.23.021704.115633

[b34] Kamogawa-Schifter Y. *et al.* Ly49Q defines 2 pDC subsets in mice. Blood 105, 2787–2792 (2005). 15598811 10.1182/blood-2004-09-3388

[b35] Pelayo R. *et al.* Derivation of 2 categories of plasmacytoid dendritic cells in murine bone marrow. Blood 105, 4407–4415 (2005). 15728131 10.1182/blood-2004-07-2529 PMC1850236

[b36] Yang G. X. *et al.* CD4- plasmacytoid dendritic cells (pDCs) migrate in lymph nodes by CpG inoculation and represent a potent functional subset of pDCs. J. Immunol. 174, 3197–3203 (2005). 15749849 10.4049/jimmunol.174.6.3197

[b37] Segura E., Wong J. & Villadangos J. A. Cutting edge: B220+CCR9- dendritic cells are not plasmacytoid dendritic cells but are precursors of conventional dendritic cells. J. Immunol. 183, 1514–1517 (2009). 19570827 10.4049/jimmunol.0901524

[b38] Schlitzer A. *et al.* Identification of CCR9- murine plasmacytoid DC precursors with plasticity to differentiate into conventional DCs. Blood 117, 6562–6570 (2011). 21508410 10.1182/blood-2010-12-326678

[b39] Matsui T. *et al.* CD2 distinguishes two subsets of human plasmacytoid dendritic cells with distinct phenotype and functions. J. Immunol. 182, 6815–6823 (2009). 19454677 10.4049/jimmunol.0802008 PMC2749454

[b40] Zhang J. Q., Biedermann B., Nitschke L. & Crocker P. R. The murine inhibitory receptor mSiglec-E is expressed broadly on cells of the innate immune system whereas mSiglec-F is restricted to eosinophils. Eur. J. Immunol. 34, 1175–1184 (2004). 15048729 10.1002/eji.200324723

[b41] Tateno H., Crocker P. R. & Paulson J. C. Mouse Siglec-F and human Siglec-8 are functionally convergent paralogs that are selectively expressed on eosinophils and recognize 6’-sulfo-sialyl Lewis X as a preferred glycan ligand. Glycobiology 15, 1125–1135 (2005). 15972893 10.1093/glycob/cwi097

[b42] Zhang M. *et al.* Defining the *in vivo* function of Siglec-F, a CD33-related Siglec expressed on mouse eosinophils. Blood 109, 4280–4287 (2007). 17272508 10.1182/blood-2006-08-039255 PMC1885492

[b43] Chen G. Y. *et al.* Broad and direct interaction between TLR and Siglec families of pattern recognition receptors and its regulation by Neu1. Elife 3, e04066 (2014). 25187624 10.7554/eLife.04066 PMC4168287

