# Supplementary material for: Characterization of species-specific genes regulated by E2-2 in human plasmacytoid dendritic cells
Source: Sci Rep. 2015 Jul 17;5:10752. doi: 10.1038/srep10752 (PMC4505321; doi:10.1038/srep10752)
Supplement: Supplementary Information [file srep10752-s1.pdf]

Characterization of species-specific genes regulated by E2-2 in human plasmacytoid dendritic cells

Menglan Cheng[1], Xuyuan Zhang[1], Haisheng Yu[1], Peishuang Du[1], Joël Plumas[2],

Laurance Chaperot[2], Lishan Su[1,3] and Liguo Zhang[1]

From the [1]Key Laboratory of Immunity and Infection, Institute of Biophysics, University of Chinese Academy of Sciences, Beijing, China

[2]Department of Research and Development, EFS Rh ˆone-Alpes Grenoble, La Tronche, France

[3]Lineberger Comprehensive Cancer Center, Department of Microbiology and Immunology,School of Medicine, University of North Carolina at Chapel Hill, Chapel Hill, NC, USA

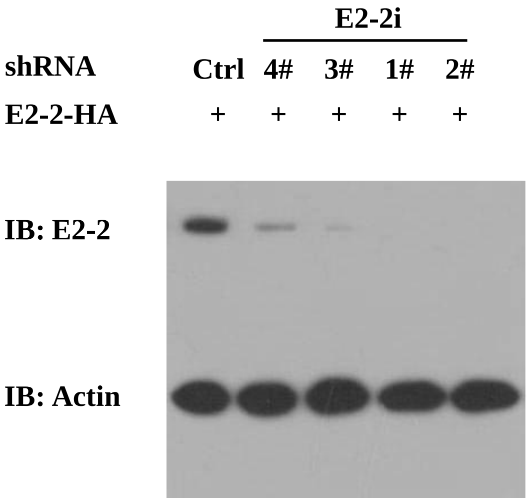

Supplementary Figure 1. E2-2 expression is silenced by shRNAs.
Immunoblotting analysis of E2-2 in lysates of 293T cells transfected with E2-2 overexpression plasmid and control shRNA or E2-2-specific shRNA 1#~4#. Actin was used as a loading control.

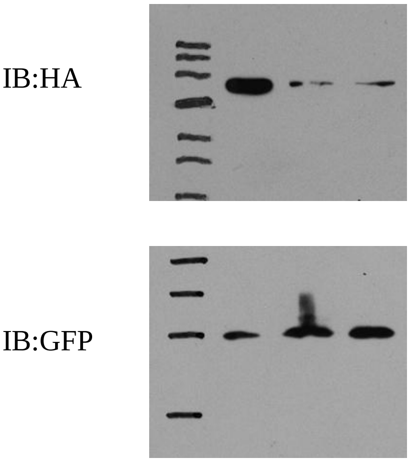

Supplementary Figure 2. Original panel for western blot from Figure 1B.

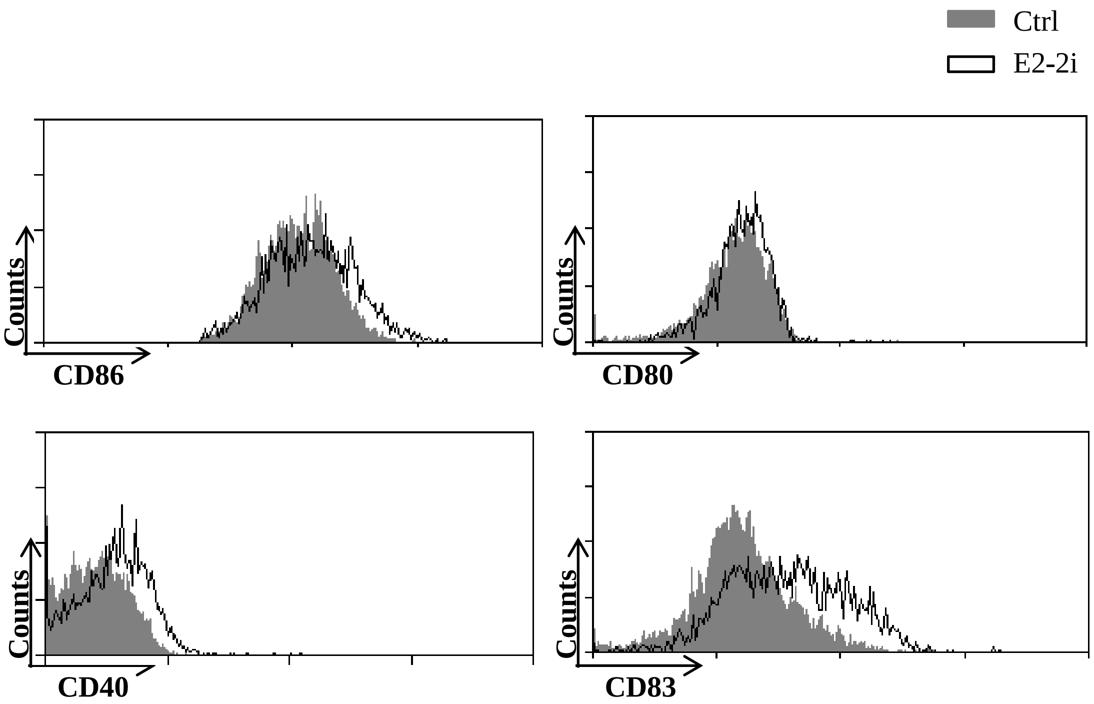

Supplementary Figure 3. Costimulatory molecules expression in GEN2.2 cells after E2-2 down-regulation.

Flow cytometry analysis of CD86, CD80, CD40 and CD83 expression on E2-2 knockdown (E2-2i) and control (Ctrl) cells.

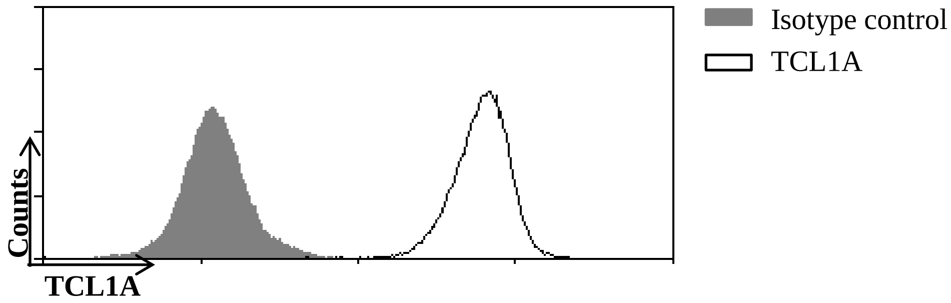

Supplementary Figure 4. TCL1A expression in GEN2.2 cells.
Flow cytometry analysis of intracellular TCL1A expression in GEN2.2 cells after cell fixation and permeabilization.
